# Extracting Cardiac Information From Medical Radar Using Locally Projective Adaptive Signal Separation

**DOI:** 10.3389/fphys.2019.00568

**Published:** 2019-05-21

**Authors:** Yu Yao, Guanghao Sun, Tetsuo Kirimoto, Michael Schiek

**Affiliations:** ^1^Translational Neuromodeling Unit, University of Zurich–ETH Zurich, Zurich, Switzerland; ^2^Graduate School of Informatics and Engineering, The University of Electro-Communications, Tokyo, Japan; ^3^Central Institute ZEA-2—Electronic Systems, Research Center Jülich, Jülich, Germany

**Keywords:** signal processing, non-linear filtering, medical radar, vital signs monitoring, cardiac signal

## Abstract

Electrocardiography is the gold standard for electrical heartbeat activity, but offers no direct measurement of mechanical activity. Mechanical cardiac activity can be assessed non-invasively using, e.g., ballistocardiography and recently, medical radar has emerged as a contactless alternative modality. However, all modalities for measuring the mechanical cardiac activity are affected by respiratory movements, requiring a signal separation step before higher-level analysis can be performed. This paper adapts a non-linear filter for separating the respiratory and cardiac signal components of radar recordings. In addition, we present an adaptive algorithm for estimating the parameters for the non-linear filter. The novelty of our method lies in the combination of the non-linear signal separation method with a novel, adaptive parameter estimation method specifically designed for the non-linear signal separation method, eliminating the need for manual intervention and resulting in a fully adaptive algorithm. Using the two benchmark applications of (i) cardiac template extraction from radar and (ii) peak timing analysis, we demonstrate that the non-linear filter combined with adaptive parameter estimation delivers superior results compared to linear filtering. The results show that using locally projective adaptive signal separation (LoPASS), we are able to reduce the mean standard deviation of the cardiac template by at least a factor of 2 across all subjects. In addition, using LoPASS, 9 out of 10 subjects show significant (at a confidence level of 2.5%) correlation between the R-T-interval and the R-radar-interval, while using linear filters this ratio drops to 6 out of 10. Our analysis suggests that the improvement is due to better preservation of the cardiac signal morphology by the non-linear signal separation method. Hence, we expect that the non-linear signal separation method introduced in this paper will mostly benefit analysis methods investigating the cardiac radar signal morphology on a beat-to-beat basis.

## Introduction

Electrocardiography (ECG) has become a universally accepted standard for measuring heart rate. However, since ECG is caused by depolarization and repolarization of the heart, it is difficult to directly asses the mechanical activity of the heart using ECG (Chan-Dewar, 2012). Recently, research has been focused on unobtrusive measurements of mechanical heartbeat activity using, e.g., ballistocardiography (BCG) or seismocardiography (Castiglioni et al., 2007; Postolache et al., 2007; Inan et al., 2009a; Pinheiro et al., 2010; Giovangrandi et al., 2011). Most of these projects aim to develop unobtrusive, long-term home monitoring systems for monitoring patients with cardiac conditions (Brüser et al., 2012, 2013; Christoph Hoog et al., 2015) or monitoring sleep quality (Paalasmaa et al., 2012, 2014).

While these applications are undoubtedly of great importance, the original intention behind the development of BCG and related modalities was to non-invasively asses the mechanical cardiac and hemodynamic activity and derive covariates of measures such as cardiac output (Starr and Noordergraaf, 1967). For these purposes, it is important to obtain a clean signal with high signal-to-noise ratio. Additionally, one must ensure that the preprocessing steps do not significantly change the morphology of the signal. Unfortunately, mechanical heartbeat signals obtained with modern systems often contain a strong respiratory signal component (Yao et al., 2012). Removing this component without altering the cardiac signal can pose a certain challenge when the respiratory movements contain high frequency components like sharp flanks (Yao et al., 2014). Additionally, the cardiac signal itself can contain high frequency components due to the influence of heart sounds (Castiglioni et al., 2011), which makes it difficult to reduce noise using simple low-pass filtering (Yao et al., 2012). For these reasons, we have previously adapted a non-linear algorithm to separate the respiratory and cardiac components from BCG recordings obtained from bed-mounted sensors (Yao et al., 2014). Using simulated data, we have shown that this non-linear method achieves superior results compared to linear filters.

In this paper, we demonstrate that this non-linear signal separation method can be adapted to mechanical heartbeat signals obtained with radar. Medical radar for non-contact vital signs acquisition is a rapidly developing modality, which faces very similar challenges to BCG, including sensitivity to movement artifacts, high variability in signal morphology and spectral overlap between cardiac and respiratory components (Sun et al., 2011a,b, 2012a, 2013, 2016; Khan and Cho, 2017).

In addition, we also address the issue of parameter estimation, which was identified as a weakness of the original non-linear signal separation method, by improving an automated parameter estimation scheme developed for BCG signals (Yao et al., 2013). This parameter estimation scheme, which, so far, has only been tested on simulated data, is applied to real world recordings for the first time. Using sensitivity analysis, we demonstrate that the parameter estimation process is robust with respect to changes in signal amplitude and settings of the algorithm.

Combining signal separation and parameter estimation, we obtain an adaptive algorithm for the extraction of the cardiac component from radar signals. Since the signal separation method from Yao et al. (2014) is based on a non-linear filtering method called locally projective noise reduction (LPNR), we call the algorithm introduced here locally projective adaptive signal separation (LoPASS).

The intended application of LoPASS is to extract and denoise the cardiac component from radar recordings during the preprocessing step prior to any analysis of mechanical cardiac activity that requires precise knowledge of the signal morphology. In order to demonstrate the advantage of LoPASS, we compare the results from preliminary analyses performed on a dataset of radar recordings which have been preprocessed with either LoPASS or linear filters. We chose linear filters for this comparison, since they are still used regularly for the extraction of the cardiac component, even by state-of-the-art beat detection algorithms (Paalasmaa et al., 2014).

Specifically, we perform two benchmark applications: First, we extract a heartbeat template from the radar signal via R-peak-synchronized averaging, showing that the LoPASS-preprocessed data exhibits much higher coherence and lower standard deviation. Then, we examine the relationship between the timing of the peaks in the radar signal and the R-T-interval. Since ventricular systole, the main cause for the deflections in the cardiac radar signal, takes place during the R-T-interval (Chan-Dewar, 2012), we expect a correlation between the timing of the main deflection in the radar signal and the R-T-interval. This correlation is more easily detected in the LoPASS signal than in the signal extracted with linear filters.

This article is structured as follows. In Section “Materials and Methods,” we describe the proposed LoPASS algorithm and investigate its robustness via sensitivity analysis. In addition, we also provide details on the data acquisition procedure. Section “Results” contains the results of the data analysis and the comparison between linear filters and LoPASS-based preprocessing. In Section “Discussion,” we will discuss our findings, and finally a conclusion.

## Materials and Methods

### Non-linear Signal Separation

The non-linear signal separation used in this paper was previously applied to the problem of extracting fetal ECG signals (Schreiber and Kaplan, 1996b) and separating the respiratory and cardiac components of BCG signals (Yao et al., 2014). It is based on the so called LPNR (Kantz and Schreiber, 2002), which is a non-linear filtering method using geometric projections in delay space to achieve reduction of in-band noise. Variations of LPNR have been used to denoise signals from a variety of applications domains, such as BCG (Yao et al., 2014), ECG (Schreiber and Kaplan, 1996a), electroencephalography (Effern et al., 2000) or natural language (Hegger et al., 2000), attesting to its generality. A detailed introduction to both the LPNR algorithm and LPNR-based signal separation can be found in Kantz and Schreiber (2002) and Yao et al. (2014). In the following, we provide a short summary of its working principle.

Deterministic signals tend to occupy a low dimensional manifold when embedded into delay space using delay embedding techniques, while noise generally spreads into all dimensions of the delay space. This property of deterministic signals, which is formally described in Takens’ delay embedding theorem (Takens, 1981), can be exploited for noise reduction, even when the signal in question is not strictly deterministic as, e.g., in the case of ECG or BCG signals. In short, future samples from a deterministic signal can be predicted from past samples:

(1) $$x_{T+1}=f(x_{T})$$

Here, *x_t_* is a delay vector containing *m* samples up to time step *t*, where m is the so-called embedding dimension:

(2) $$x_{t}={\left(x_{t-m},...,\;x_{t-1},x_{t}\right)}^{T}$$

Linearizing the function *f*(⋅), which encodes the underlying dynamics that generated the signal, one obtains an approximation, at the current location *x*_0_, to the manifold in delay space occupied by the deterministic signal:

(3) $$x_{T+1}=x_{0}+A(x_{T}-x_{0})$$

Since the manifold is restricted to a lower-dimensional subspace, matrix *A* encodes the dimensions into which the signal extends, at least locally around *x*_0_. Hence, projecting onto the subspace spanned by the columns of *A* will reduce noise, under the assumption that the space perpendicular to *A* is occupied only by noise.

The principle outlined above was formalized by Grassberger et al. (1993), who also showed that the subspace spanned by *A* can be estimated given only noisy data and no prior information about the dynamics of the signal, by calculating the principal eigenvector of the set *Y_t_* of noisy delay vectors ***y*** within a small region of size ε around the current delay vector ***y***_*t*_:

(4) $$Y_{t}=\{y:||y-y_{t}||<\varepsilon \}$$

**FIGURE 1:**
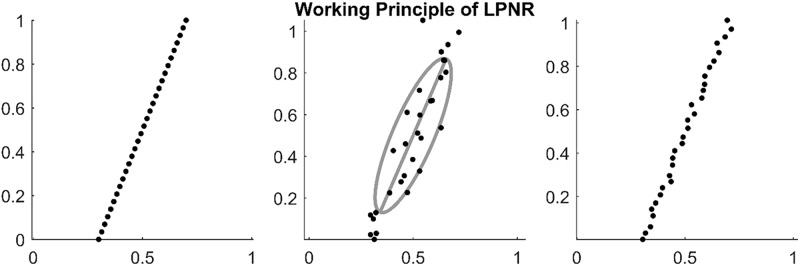
**(Left)** Embedding a deterministic signal results in delay vectors on a low dimensional manifold. **(Middle)** Noise causes the delay vectors to spread into all dimensions of the delay space. However, the original manifold can be recovered by calculating the main eigenvector of the delay vectors within a region of size ε as shown by the gray ellipse (in this plot ε = 1). **(Right)** By projecting on the main eigenvector (principal axis of the gray ellipse), one can reduce the noise level.

In Figure 1, we illustrate an example of LPNR denoising a signal by projecting it onto the one-dimensional manifold in a two-dimensional delay space. Critically, the choice of ε determines the intensity of noise reduction and the common recommendation is to choose the value of ε equal to the expected peak-to-peak amplitude of the noise process (Kantz and Schreiber, 2002).

**FIGURE 2:**
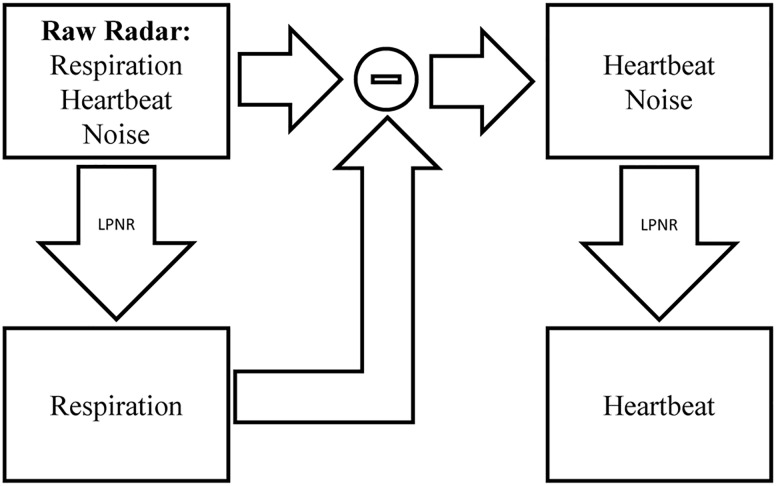
Signal separation using LPNR. The input signal (here radar) containing respiration, heartbeat and noise is filtered using LPNR with parameter settings such that heartbeat and noise are removed (specifically, ε approximately equals the heartbeat amplitude). Subtracting respiration from the input and applying LPNR again, with parameter settings such that noise is removed, extracts the heartbeat component.

This uncommon method of specifying the noise characteristic, combined with the fact that LPNR operates in delay space as opposed to frequency domain, enables this method to perform noise reduction even if the noise process has a similar spectrum as the signal (Schreiber, 1999). To separate two signal components *s*_1_ and *s*_2_ with overlapping spectra, one simply applies LPNR to remove one of the components, say *s*_1_ (usually, the faster and smaller component), in order to obtain an estimate of the other component *s*_2_. This is achieved by setting ε equal to the peak-to-peak amplitude of *s*_1_, which is typically obtained by visual inspection. Subtracting the estimate of *s*_2_ from the raw input signal then yields an estimate of the first component *s*_1_ (Schreiber and Kaplan, 1996b). This process is illustrated in Figure 2, where *s*_1_ corresponds to the heartbeat and *s*_2_ to the respiration signal.

By design, LPNR operates on the entirety of the input data, meaning it is technically not a filter but a smoother. However, fast online approximations to the LPNR algorithm have also been developed (Schreiber and Righter, 1999).

### Adaptive Parameter Estimation

One of the main weaknesses of LPNR-based methods is the choice of the parameters, and specifically the choice of ε. The general approach is to choose this parameter via visual inspection (Kantz and Schreiber, 2002). While this approach might work in applications where the analysis is done offline (see, e.g., Schreiber and Kaplan, 1996b; Effern et al., 2000), it is not feasible for systems that are designed to operate autonomously. This problem is most relevant for mechanically measured heartbeat signals acquired by unobtrusive home monitoring systems, because the signals tend to display non-stationary behavior due to the long observation periods. Additionally, these systems most likely employ some kind of automatic gain control to deal with the uncertainty of the measurement environment. Hence, a fixed parameter setting will not work for such systems.

We have previously introduced an algorithm for estimating a suitable value for ε for the separation of BCG component (Yao et al., 2013). Critically, we have shown using simulated BCG recordings that the estimate for ε scaled correctly with the amplitude of the cardiac component. However, the parameter estimator was not tested on real data. In this paper, we introduce a new adaptive parameter estimation scheme based on a similar principle as the one proposed in Yao et al. (2013) and apply the scheme to real world recordings of medical radar data.

Similar to our approach in Yao et al. (2013), we collect the maximum amplitude difference Δ*a_t_* within slices **Δ*s***_*t*_ of the high-pass-filtered signal ***s*** = (*s*_0_,…, *s_t_*,…, *s_T_*). The slice length τ is chosen to be slightly longer than the expected beat-to-beat interval (τ = 1.5s in the current implementation):

(5) $$\Delta s_{t}=(s_{t},...,s_{t+\tau })$$

(6) $$\Delta a_{t}=\max(\Delta s_{t})-\min(\Delta s_{t})$$

Next, we calculate the median *m_a_* of the amplitude differences from all slices:

(7) $$m_{a}=median(\{\Delta a_{t}:t=0s,1.5s,3s,\ldots \})$$

The median of a set *X* of values *x_n_* is defined as the value that is, at the same time, smaller or equal and larger or equal than half of all values in *X*:

(8) $$median(X)=x_{m}\;with\;x_{m}\;\in X\;such\;that\;|\{x_{n}:x_{n}\leq x_{m}\}|=|\{x_{n}:x_{n}\geq x_{m}\}|=[N/2]$$

The estimate for ε is then taken as 1.5 times the median:

(9) $$\varepsilon :=f_{sc}m_{a},\;with\;f_{sc}=1.5$$

**FIGURE 3:**
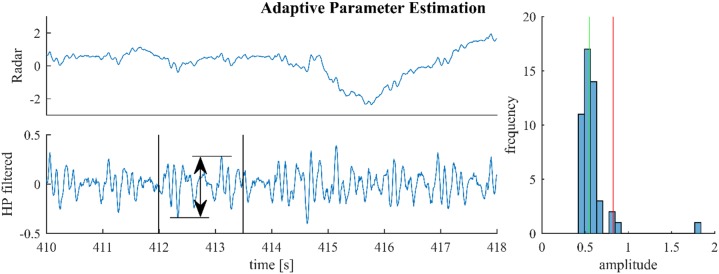
For parameter estimation the raw signal (upper left) is high-pass-filtered and the maximum altitude difference in slices of 1.5 s are collected throughout the signal. One such slice is shown in the lower left plot. The median of these amplitude differences is multiplied with 1.5 and used as the estimate (green and red lines, right plot).

Figure 3 illustrates this process for a segment from a medical radar recording containing both cardiac and respiratory signal components. On first sight, scaling the median by an arbitrary factor seems to be inferior to using the 70% quantile and rejecting the remaining 30% as outliers, as done in Yao et al. (2013). However, the median is generally much more stable than the tails of a distribution. Notably, the value of the 70% quantile might change considerably depending on the presence or absence of outliers. In the example from Figure 3, the histogram in the right plot shows that the outlier at 1.8 is correctly rejected; although, the estimator also rejects a small part of the signal (the part beyond the red line and below 1).

In practice, we apply parameter estimation to epochs of 1 min. The estimate of ε is then used for LPNR-based signal separation on the given epoch and the whole procedure of estimation and separation is repeated for each epoch. Combining the adaptive parameter estimation method with LPNR-based signal separation eliminates the need to manually choose the parameter ε and results in an algorithm that can adapt itself to signals with non-stationary amplitudes. Hence, we call this algorithm locally projective adaptive signal separation (LoPASS).

Previously, we have shown that LPNR-based signal separation is relatively robust against small misspecifications of the ε parameter (Yao et al., 2014). Here, we perform a sensitivity analysis in order to quantify the robustness of LoPASS toward the choice of *f_sc_*. For this purpose, we vary *f_sc_* between 1 and 2 and compare the output of LoPASS to the output obtained with the standard setting of *f_sc_* = 1.5. Specifically, we calculate the relative variance of the difference signal:

(10) $$e_{rel}=\frac{var(c_{x}-c_{1.5})}{var(c_{1.5})},\;with\;x=1,...2$$

**FIGURE 4:**
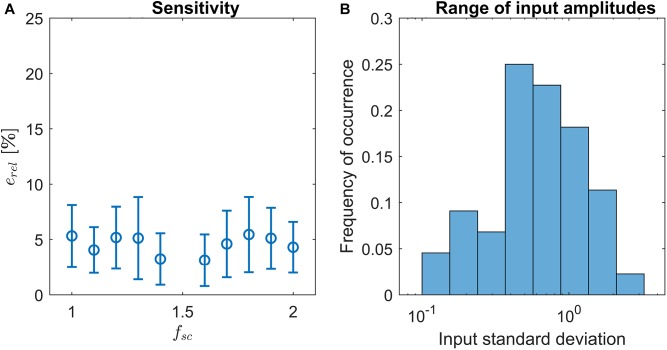
**(A)** Result of sensitivity analysis: Varying the scaling factor *f*_sc_ by up to ±50% results in a relative change of the output *e*_rel_ (blue circles) by around 5% on average. Error bars indicate one standard deviation. **(B)** Histogram over standard deviations of radar recordings. Note the logarithmic scale of the *x*-axis.

Here, *c_x_* denotes the output of LoPASS obtained with *f_sc_* = *x*. The result, which is shown in Figure 4A, indicates that changing *f_sc_* by up to 50% only results in changes of the output of about 5% on average. The histogram of input signal amplitudes in Figure 4B shows that this result is not simply due to a lack of variation in the input signals. Hence, we conclude that LoPASS is extremely robust with respect to the choice of *f_sc_*.

### Medical Radar

In this paper, we demonstrate that LPNR-based signal separation can be applied to recordings obtained from medical radar. Medical radar has been proposed as contactless vital signs estimation method for use in infection screening systems (Sun et al., 2011a,b, 2012a,b, 2013; Yao et al., 2016) and depression screening systems (Sun et al., 2016). It works by recording the motion of the body surface induced by left ventricular ejection and aortic blood flow. When measured on the chest wall it is also known as apexcardiography (Lin et al., 1979).

**FIGURE 5:**
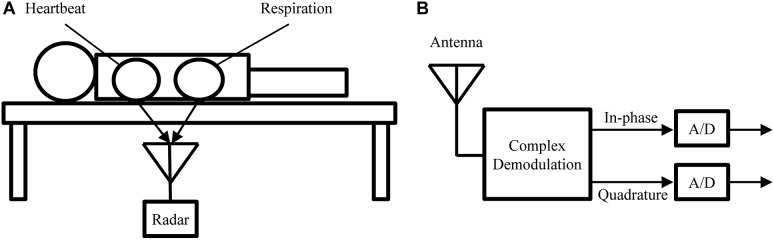
**(A)** Measurement setup used to obtain the experimental recodings analyzed in this paper. **(B)** Block diagram of the radar receiver.

Similar to BCG, radar can be used in a bed-mounted configuration as shown in Figure 5. Such a setup is suitable for both home monitoring application or scientific investigations involving the mechanical heartbeat activity. Compared to BCG, which uses pressure sensors, or SCG, which uses body-mounted accelerometers, radar has the advantage that it is a truly contactless modality. This is of importance in applications where direct contact is undesirable, e.g., in infection screening systems (Sun et al., 2012a). Additionally, the radar receiver can be more easily repositioned to adjust for differences in body size or posture, while BCG sensors are generally fixed to the frame of the bed.

### Data Acquisition

In this paper, we perform a preliminary analysis on recordings from medical radar obtained in a controlled laboratory experiment. The main goal of this analysis is to assess the ability of LoPASS to extract the cardiac components from radar recordings. Figure 5A shows the measurement setup. Several recordings, each 10 min long, were obtained for each subject, with the radar placed at different positions under the bed, while the subject was lying on the bed in a supine position and breathing normally. The cohort consisted of a total of 10 university students without known cardio-respiratory diseases (9 males and 1 female, average age: 22 years). Examples of raw data from 10 subjects are provided in Supplementary Table S1. These examples correspond to the part of the dataset that was used for the analysis presented in “Results” Section. This dataset has not been previously published. In this study, we focus on extracting the cardiac component. Therefore, the radar was located around the apex of the heart (left fifth intercostal space) with a distance of 3–5 cm.

Since the 24-GHz radar system (SHARP, DC6M4JN3000, Japan) used in the experiments was a prototype, which lacked automatic gain control, the amplification of the system was manually adjusted for each recording, with signal amplitudes differing by more than one order of magnitude across recordings (see Figure 4B). Hence, the recordings in the dataset poses sufficient variability to challenge the ability of LoPASS to adapt itself to substantial variations in signal amplitude and morphology.

The experiment was approved by the ethics committee of the University of Electro-Communications, Tokyo, Japan. All participants were fully informed of the purposes and experimental procedures before they gave their written consent to participate.

Since the objective of the analysis is to assess the extraction of the cardiac component, we visually inspected all recordings and selected one representative, artifact free segment per subject. By excluding movement artifacts from the analysis, we focus on the ability of the separation algorithm to distinguish between respiration and heartbeat and minimize the possibility that our result might be biased by potential differences in resistance against artifacts between linear and non-linear preprocessing methods. Following this approach, we do not assess the resilience of LoPASS to movement artifacts. However, since it is known that movement artifacts assert a strong influence on mechanical heartbeat signal (Inan et al., 2010a,b), we think that the most promising approach is to perform artifact rejection using a separate algorithm optimized for this task, before performing signal separation. Hence, in the current analysis, we focus only on cardiac component extraction from artifact free recordings.

## Results

In this section, we present the results of our preliminary analysis. The main goal of this analysis is to compare LoPASS-based preprocessing to conventional linear filter-based preprocessing. Therefore, all analysis steps are carried out on two sets of data: (i) the artifact free radar recordings which were preprocessed using LoPASS and (ii) the same recordings preprocessed with linear filters. A secondary goal of this analysis is to assess the viability of radar as an unobtrusive and non-invasive modality for investigating medical questions of interest involving the mechanical heartbeat activity. For these reasons, we performed two benchmark applications: template construction and peak timing analysis. The following sections describe the analysis performed and the result.

The raw data used in the analysis presented in this section is released in the Supplementary Table S1.

Additional data not included in the Supplementary Table S1 was used for the sensitivity analysis in Section “Adaptive Parameter Estimation.”

### ECG Assisted Template Construction

**FIGURE 6:**
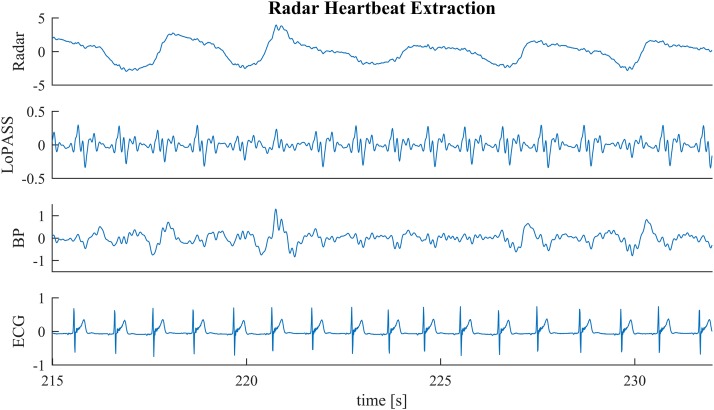
A sample segment from the radar recording (first row) with cardiac components extracted via the LoPASS algorithm (second row) and conventinal bandpass filtering (third row). The simultaneously recorded ECG reference is also shown (fourth row).

In the first benchmark application, we use the R-peaks of a simultaneously recorded ECG signal to construct a template of the cardiac radar component. Template construction is an important step in the analysis of mechanical heartbeat signals in both classical (Starr and Noordergraaf, 1967) and modern (Inan et al., 2009b) analysis approaches, as well as in some heart rate estimation methods (Paalasmaa et al., 2014). However, constructing templates is sensitive to the consistency of the signal morphology, which can be affected by non-stationarities (e.g., change of posture) but also by the influence of high frequency parts of the respiratory component on the heartbeat signal. An example of such a situation can be observed in Figure 6 at 218 s, at 227 s, and at 231 s. Although, the radar signal (Figure 6 first row) does not contain any visible artifacts at these time points, the upward flank of the respiratory component contains high frequency parts that remain after bandpass filtering and cause visible artifacts in the heartbeat signal (third row). Notably, the cardiac component extracted using LoPASS is not visibly affected (Figure 6 second row).

In order to quantify the resistance of LoPASS to these kinds of high frequency respiratory interference and its advantage compared to bandpass filtering, we extract the cardiac component from artifact free segments of the radar signal with both LoPASS and linear filters. The linear filters consisted of a finite impulse response (FIR) low-pass filter with order 128 and a cutoff frequency of 10 Hz, which was used to remove high frequency noise components. In addition, an FIR high-pass filter with order 256 and cutoff frequency of 0.75 Hz was used to remove the respiratory component. Using separate filters for these two tasks allowed us to adapt the filter steepness to each task separately. Furthermore, since the exact heart rate is not known in advance, we chose conservative cutoff frequencies resulting in a relatively wide bandwidth, in order to account for changes in heart rate.

**FIGURE 7:**
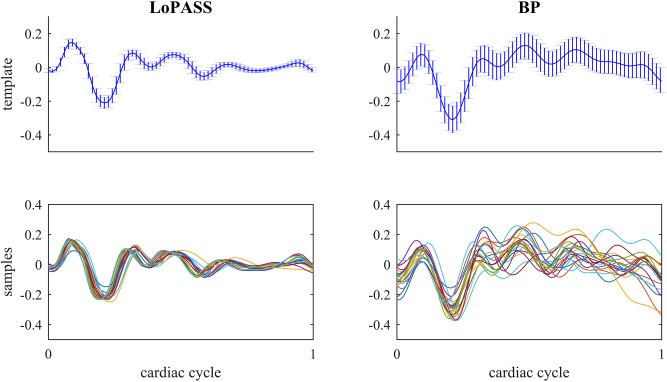
Top row: Template of mechanical heartbeat calculated from the cardiac radar signal extracted with LoPASS **(Left)** and linear filters **(Right)** from the radar recording of subject 1. Errorbars show the standard deviation. Bottom row: Twenty samples chosen at random from the pool of all samples used to calculate the template in the top row. In all plots, the *x*-axis shows the time relative to the cardiac cylce, where 0 corresponds to the occurrence of the previous R-peak and 1 corresponds to the occurrence of the next R-peak.

For both separation methods, the resulting cardiac component was segmented using the R-peaks detected in the simultaneously recorded ECG reference, and a template of the mechanical heartbeat signal was calculated by resampling and averaging. In addition, we also calculated the standard deviation, which quantifies the deviation from the template. This procedure was performed for each subject individually. The resulting templates for subject 1 are shown in Figure 7 along with 20 randomly chosen samples. From visual inspection, it is evident that the cardiac component extracted with LoPASS is much more consistent than the cardiac component extracted with linear filters. This impression is also confirmed quantitatively by the standard deviation listed for the all 10 subjects in Table 1. Since value of the standard deviation depends on the position in the cardiac cycle, we calculated the mean standard deviation across the whole template. The mean standard deviation for the cardiac component extracted by the linear filter is consistently more than twice as large as the mean standard deviation of the LoPASS-extracted cardiac signal. Note that the absolute value of the standard deviation also depends on the amplitude of the radar signal and thus cannot be compared across subjects.

**Table 1:** Average standard deviations across the templates calculated based on LoPASS and linear filter-extracted cardiac signal and their ratio.

Subject no.	Mean standard deviation LoPASS	Mean standard deviation BP	Ratio std BP/std LoPASS
1	0.0235	0.0758	3.22
2	0.0327	0.0743	2.27
3	0.1104	0.2645	2.40
4	0.0253	0.0677	2.68
5	0.0939	0.1988	2.12
6	0.0392	0.0810	2.07
7	0.2198	0.5436	2.47
8	0.0206	0.0569	2.76
9	0.0243	0.0617	2.53
10	0.0735	0.2575	3.50
Average ratio std BP/std LoPASS across all subjects:			2.6


*Only the ratio is comparable across subjects, since the standard deviations themselves depend on the subject specific amplitude of the raw signals.*

This result demonstrates that LoPASS-based cardiac component extraction leads to both visually and quantitatively more consistent templates as opposed to linear filter-based cardiac signal extraction. Judging from the samples shown in the lower row of Figure 7, this effect is not only due to a superior baseline removal achieved by LoPASS, but also due to a more consistent signal morphology, which is especially evident in the second half of the cardiac cycle. This has profound consequences for applications which rely on detailed investigation of the cardiac signal morphology on a beat-to-beat basis. One example of such an application is introduced in the following section.

### Peak Timing and R-T-Interval

In the second benchmark application, we look at the relationship between the timing of the major peaks in the cardiac radar signal measured with respect to the R-peak of the simultaneously recorded ECG and the R-T-interval. The R-peak in the ECG signal marks the depolarization of the ventricle, while the T-wave marks the repolarization (Chan-Dewar, 2012). However, it is the mechanical activity of the heart which causes the cardiac component observed in radar or BCG recordings. Specifically, it is thought that the ejection of blood from the left ventricle causes the first major peak of each cardiac cycle in the BCG signal (Starr and Noordergraaf, 1967). With respect to the electrical cardiac cycle, ventricular ejection takes place between the R- and T-peak (Chan-Dewar, 2012). Variations in the duration of ventricular systole are likely to affect the timing of ejection and T-peak in a similar way. Hence, we conjecture that the timing of the first major peak of each cardiac cycle in the cardiac radar component is correlated with the R-T-interval.

**FIGURE 8:**
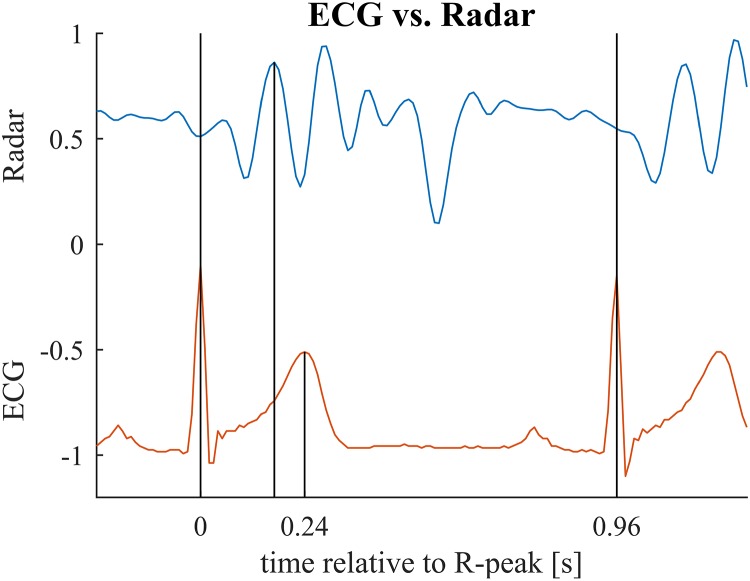
Relevant time points for the second benchmark application illustrated for one cardiac cycle. First vertical bar indicates the ECG R-peak (beginning of the cardiac cylce). Second bar marks the main peak in the radar signal (maximum mechanical activity). Third bar marks the T-peak (repolarization). In the benchmark application, we compare the interval between first and second bar to the interval between first and third bar. Note that these two intervals relate to different events in the cardiac cylce and hence, are not identical but are presumed to be correlated with each other. See Table 3 for mean and standard deviations of the intervals for each subject.

To confirm our conjecture, we detect the main peak in the cardiac radar signal for each cardiac cycle and calculate the correlation coefficient between the R-T-interval and the interval between R-peak and main peak of the radar signal (see Figure 8 for an example). Subsequently, we perform statistical testing whether the correlation coefficient differs significantly from zero. This procedure is performed separately for each subject and each preprocessing method (i.e., LoPASS and linear filtering) leading to a total of 20 *p*-values summarized in Table 2. Choosing a confidence level of 2.5%, we reject the null-hypothesis of zero correlation between the R-T-interval and the R-radar-interval for nine out of 10 subjects based on the cardiac radar signal extracted with LoPASS. However, when extracting the cardiac radar signal with linear filtering (with same filter parameters as in the first benchmark application), the null-hypothesis is rejected only for six of the 10 subjects.

**Table 2:** *P*-values for testing the significance of the correlation between R-T-interval and R-radar-interval.

Subject no.	*P*-values for LoPASS based preprocessing	*P*-values for linear filter-based preprocessing
1	3.7 × 10^-10^	1.2 × 10^-5^
2	1.9 × 10^-5^	8.7 × 10^-4^
3	1.1 × 10^-10^	5.8 × 10^-2^
4	1.2 × 10^-11^	1.5 × 10^-2^
5	6.4 × 10^-5^	5.6 × 10^-2^
6	2.0 × 10^-7^	2.9 × 10^-4^
7	4.9 × 10^-3^	8.6 × 10^-3^
8	6.5 × 10^-1^	7.4 × 10^-1^
9	2.6 × 10^-6^	3.9 × 10^-7^
10	2.2 × 10^-2^	2.6 × 10^-1^
Significance level: 2.5%	9 out of 10 significant	6 out of 10 significant


*The null-hypothesis states that the correlation between the two intervals is zero.*

Table 3 summarizes the mean and standard deviation of the R-T-interval and the R-radar-interval for all subjects and both preprocessing methods, as well as the correlation between the two intervals.

**Table 3:** Mean and standard deviation of R-T-interval and R-radar-interval and correlation between the two intervals for all subject separated by preprocessing method (LoPASS vs. linear filtering).

Subject no.	R-T-interval: mean (SD) [ms]	R-radar-interval for LoPASS: mean (SD) [ms]	Correlation between intervals for LoPASS	R-radar-interval for linear filter: mean (SD) [ms]	Correlation between intervals for linear filter
1	233 (4.5)	75 (5.9)	37%	184 (7.5)	21%
2	256 (4.9)	109 (4.7)	47%	298 (5.7)	35%
3	212 (4.2)	86 (6.3)	34%	277 (9.2)	9%
4	247 (9.2)	94 (7.5)	40%	275 (6.1)	12%
5	236 (4.9)	280 (7.1)	45%	282 (7.2)	27%
6	299 (4.3)	274 (5.8)	27%	100 (7.5)	18%
7	241 (6.6)	180 (6.7)	23%	195 (12.7)	21%
8	267 (7.5)	539 (26)	-8.4%	551 (38.5)	-15%
9	271 (8.0)	174 (6.0)	51%	182 (7.5)	55%
10	233 (5.4)	173 (8.4)	17%	171 (9.9)	6%
Overall	248 (30.0)	157 (91.6)	31.3% (average)	208 (77.0)	18.9% (average)


*Note that the standard deviation of the overall data mostly reflects the inter-subject variability.*

In summary, using LoPASS-based preprocessing, we observe consistent results across almost all subjects (i.e., highly significant correlation between R-T-interval and R-radar-interval). However, using linear filter-based preprocessing, the results are inconsistent with only about half of all subjects exhibiting significant correlation.

## Discussion

The results presented in the previous section demonstrate that extracting the cardiac component of the radar signal using LoPASS offers improved coherence as compared to using conventional linear filtering. Specifically, we have shown that using LoPASS leads to more coherent templates, which could benefit, for example beat detection algorithms relying on template matching techniques. Additionally, in our second benchmark application, we discovered a significant correlation between the R-T-interval in the ECG and the R-radar-interval. This suggests that the major deflections of the cardiac radar signal might indeed be closely related to the mechanical oscillations caused by ventricular ejection. Traditionally, the gold standard for measuring ventricular ejection timing is to detect the first and second heart sound using phonocardiography (Lin et al., 1979; Chan-Dewar, 2012). In future experiments, we will obtain simultaneous phonocardiograms and compare these with the cardiac radar component. If the relationship between radar signal and ventricular ejection can be confirmed, medical radar might be used as an unobtrusive and non-invasive alternative for estimating ventricular ejection timing.

Note that here, we do not claim to have established a connection between the radar signal and ventricular ejection. Instead, we have shown that using LoPASS as a non-linear preprocessing technique uncovered an interesting correlation between medical radar and ECG, which points toward promising avenues for future studies. Importantly, a consistent result across all subjects was only observed for the LoPASS-extracted cardiac radar component, suggesting that a faithful extraction of the signal morphology is necessary for such an approach to succeed.

One important limitation of this study is the small size of the dataset and the lack of heterogeneity in the cohort. Hence, it is difficult to draw any medically relevant conclusions from these results which generalize to the entire population. However, drawing medical conclusions is not the intention of this paper. Instead, this paper aims to introduce LoPASS, and to demonstrate that (i) it can be applied to medical radar recordings and that (ii) it outperforms linear filters on the benchmark applications reported here. Solving these questions for the dataset at hand provided valuable insight which, in addition to demonstrating the performance of LoPASS, will help to design future studies that overcome the limitations of the current dataset.

The improvements due to using LoPASS come at the cost of an increase in computational complexity as LPNR is significantly slower than linear filters (Yao et al., 2014), which might preclude the use of LoPASS in applications with limited computational resources (e.g., embedded systems). This problem can be mitigated by applying LoPASS to short epochs of the input data. Using this technique with an epoch length of 1 min, our MATLAB implementation of LoPASS requires a computation time which is less than 20% of the duration of the input signal. Hence, we are able to achieve quasi-online performance, albeit at the cost of a delay equal to the length of one epoch. Additional speed-ups are possible by porting the algorithm to a faster language like C. Alternatively, fast online approximations to the LPNR algorithm that avoid the delay of the epoch-based approach have also been developed. These algorithms work by storing and reusing intermediate results and are described in detail in Schreiber and Righter (1999).

One further consideration is given by applications involving average signal characteristics, like, for example the calculation of mean heart rate or mean respiration rate. For these applications, we do not expect a significant improvement from the use of LoPASS, since they do not make use of the exact morphology of the signal. However, for applications relying on the signal morphology and especially for analysis methods investigating the signal on a beat-to-beat basis, applying LoPASS for cardiac component extraction can lead to significantly improved results as demonstrated in the Section “Peak Timing and R-T-Interval.”

Finally, it is worth pointing out that since the amplification of the system was manually adjusted for each recording and since the position of the radar differed between recordings, the amplitudes of the cardiac and respiratory components of the radar signals differed by more than one order of magnitude between recordings (as indicated in Figure 4B). Hence, the adaptive parameter estimation scheme introduced in this paper was instrumental in obtaining the results reported here.

## Conclusion

In this paper, we have adapted a non-linear signal separation algorithm to extracting the cardiac component from medical radar recordings. Additionally, we have augmented this separation scheme with an adaptive parameter estimation method, which addresses the weakness of having to estimate the parameter for LPNR via visual inspection. The resulting locally projective adaptive signal separation (LoPASS) algorithm is best suited as a preprocessing step for analysis methods where preserving signal morphology is critical. Using two benchmark applications for radar recordings, we have demonstrated the superiority of LoPASS as compared to linear filters. In future projects, we will perform more detailed analyses of the cardiac radar signal. These will include comparisons of the cardiac radar signal to phonocardiograms, with the prospect of establishing radar as an unobtrusive and non-invasive estimator for ventricular ejection timing.

## Ethics Statement

The experiment was approved by the ethics committee of the University of Electro-Communications. All participants were fully informed of the purposes and experimental procedures before they gave their written consent to participate.

## Author Contributions

YY, GS, TK, and MS conceptualized and designed the study. GS contributed to the acquisition of data. YY and GS analyzed and interpreted the data. YY, GS, and MS drafted the manuscript. All authors have approved the final version to be published.

## Conflict of Interest Statement

The authors declare that the research was conducted in the absence of any commercial or financial relationships that could be construed as a potential conflict of interest.

## Supplementary Material

TABLE S1 Raw data from 10 subjects used for the benchmark applications presented in “Results” Section.
